# Preparation and Structure Characterization of High-Value *Laminaria digitata* Oligosaccharides

**DOI:** 10.3389/fnut.2022.945804

**Published:** 2022-07-07

**Authors:** Kit-Leong Cheong, Jia-Kang Li, Saiyi Zhong

**Affiliations:** ^1^Guangdong Provincial Key Laboratory of Aquatic Product Processing and Safety, College of Food Science and Technology, Guangdong Ocean University, Zhanjiang, China; ^2^Department of Biology, College of Science, Shantou University, Shantou, China

**Keywords:** laminaran, oligosaccharide, *Laminaria digitata*, size exclusion chromatography, biological activity

## Abstract

Algae-derived marine oligosaccharides have been reported to be promising bioactive compounds because of their various properties with health benefits and potential significance in numerous applications in industrial biotechnology. In this study, laminaran oligosaccharides (LOs) with varying degrees of polymerization were obtained through partial acid hydrolysis of laminaran derived from *Laminaria digitata*. Based on response surface methodology, the optimum LOs yield was obtained for acid hydrolysis laminaran at a hydrolysis time of 55 min, temperature of 71°C, and acid concentration of 1.00 mol/L. The size-exclusion resin Bio-Gel P-2 was considered to be a better option for LOs purification. The structure of the purified oligosaccharides was analyzed through mass spectrometry and nuclear magnetic resonance. They demonstrated the main oligosaccharide structure corresponding to the connection of glucose with β-D-Glc*p*-(1→3)-β-D-Glc*p*, which was identified as laminaribiose (DP2), laminaritriose (DP3), laminaritetrose (DP4), and laminaripentaose (DP5). LOs demonstrate excellent antioxidant activities, as evidenced from their reactions with oxidizing free radicals, 1, 1-diphenyl-2-picryl-hydrazyl, and 2, 2′-azino-bis (3-etilbenzotiazoline-6-sulfonic acid) radicals. LOs exhibited a prebiotic effect on the growth of *Bifidobacterium adolescentis* and *Lactobacillus plantarum*. Therefore, we propose the development of LOs as natural antioxidants and prebiotics in the functional food and pharmaceutical industries.

## Introduction

Marine algae, an important component of the coastal marine ecosystem, are a good source of phenolic compounds, carotenoids, vitamins, minerals, peptides, proteins, and polysaccharides (1, 2). *Laminaria digitata* is a large brown alga in the family Laminariaceae that is extensively cultivated in China, where it is traditionally used for human consumption because of its excellent taste and nutritional value. It is an important and commercially valuable resource for food, animal fodder, and pharmaceuticals (3). *L. digitata* is rich in polysaccharides known as laminaran, which has garnered increasing attention from scientists. This macromolecule has been demonstrated to have potential to improve human health and impart various biological functions such as antitumor, anticoagulant, antiallergic, antiviral, antitumor, and antibacterial properties (4, 5).

Laminaran is a β-glucan with a backbone of β-1, 3-glycosidic linkages and is branched with small amounts of β-1, 6-glycosidic linkages. In general, the bioactivity of laminaran is closely correlated to its molecular weight, linkage type, degree of branching, and content. Laminaran with large molecular weight and size are usually poorly soluble, and it is difficult to span the membranes to exert their biological effects (6). Laminaran oligosaccharides (LOs) are known to have a low molecular weight with a degree of polymerization (DP) in the range of 2–10, and in recent years, they have garnered increasing interest because of their potential biological activities. Choi et al. reported that laminaran was degraded by gamma irradiation to produce low-molecular-weight laminaran, which exhibited better 2, 2-diphenyl-1-picrylhydrazyl (DPPH) radical-scavenging activity and protection of lipid peroxidation compared to laminaran (7). Wu reported that oligosaccharides derived from *L. japonica* exhibited high hydroxyl radical scavenging activity (91.31%) at a concentration of 100 μg/mL (8). Antioxidant activity is an important physiological role of functional foods. Consumption of antioxidants helps scavenge free radicals in human biological systems and could reduce the incidences of chronic diseases such as heart disease and cancer (9). Purified oligosaccharides can be identified more accurately by assessing their activities and demonstrating their pharmacological mechanism (10). However, the biological activities of LOs have been investigated with the crude LOs fraction in recent studies, whereas purified LOs have seldom been performed. Further studies are required to clarify the antioxidant activity of purified LOs.

The production of marine algae oligosaccharides through hydrolysis generally requires the extraction of polysaccharides from marine algae, followed by depolymerization. The degradation products prepared by different methods differed in the structures. Enzymatic method is highly specific for oligosaccharide production but the relatively high costs of enzyme production, which makes them unfavorable from an economic point of view. Chemical methods for the preparation of marine algae oligosaccharides are considered useful and widely used because they are relatively simple, inexpensive, and controllable (11). The mixture of LOs derived from chemical methods needs to be further purified because the purity of oligosaccharides is also important for the potentiation of their physiological effects and to obtain a marketable product. Purification of LOs with varying DP remains a challenge because of their high polarity and laborious procedures for obtaining high-purity oligosaccharides. Thus far, there have been only a few reports on the preparation and purification of LOs. However, pure LOs are necessary for quantification analysis and clarification of their related biological activity mechanisms for the development of marine functional food, drugs, and related products (12). Therefore, it is important to develop a high-resolution, automated, and simple procedure for the preparation and purification of LOs. Fast protein liquid chromatography (FPLC) is generally applied only for protein and peptide purification because it only has an ultraviolet detector. Because oligosaccharides do not absorb ultraviolet light, auto-peak collection may be difficult (13). Traditionally, colorimetric assays have been used for offline detection of oligosaccharide fractions (14). However, they have several drawbacks such as being laborious, slow, and consuming large amounts of hazardous reagents. Recently, FPLC coupled with the refractive index detection (RID) method has been successfully applied in the separation and purification of oligosaccharides, and has been proven to be highly automated with a high resolution (15).

In this study, LOs were produced from *L. digitata* polysaccharides through partial acid hydrolysis. LOs with varying DPs were purified by an FPLC system coupled with a refractive index detector, which is usually used to detect carbohydrates and fulfill the automated collection of the oligosaccharide fraction. The effects of DP on antioxidant properties, including DPPH and ABTS radical scavenging activities, and the prebiotic activities of the purified LOs were investigated.

## Materials and Methods

### Materials and Chemicals

Sephadex G-10 and Sephadex LH-20 were purchased from GE Healthcare (Uppsala, Sweden). Bio-gel P-2 extra fine was purchased from the Bio-Rad company (California, United States). HPLC-grade acetonitrile and trifluoroacetic acid (TFA) were purchased from Aladdin Chemical Co. (Shanghai, China). The DPPH and ABTS were acquired from Sigma-Aldrich. The *Bifidobacterium adolescentis* and *Lactobacillus plantarum* were purchased from China Center of Industrial Cultural Collection. All other chemicals and reagents were of analytical grade.

### Preparation of Laminaran

The dried *L. digitata* was homogenized in a blender. The powder was first extracted with methanol/ dichloromethane/ water (4:2:1; *v/v/v*) to remove small molecular weight compounds, color matter, and lipids (16). The laminaran was obtained according to previous report with minor modifications (17). The residue was collected by centrifugation at 4,000 × g for 10 min. After dried in an oven at 50 °C until a constant weight, the residue was extracted with hot water in a ratio of 1:40 (*w/v*, g/mL) at 90°C for 2 h. After cooling to room temperature, the aqueous extract was filtrated and then added three times volume of 95% (*v/v*) ethanol, and the precipitated was collected through centrifugation (3,500 × g, 10 min). The pellet was re-dissolved in hot water and left overnight 4°C, the supernatant thus obtained was centrifuged to remove the insoluble matter. Subsequently, the supernatant was then mixed with aqueous calcium chloride (2%, *w/v*) and centrifuged at 3,500 × g for 10 min. The precipitates dissolve in water and dialysis (molecular weight cut-off 500 Da) against water. Further purification involved the separation of fractions loaded on to solid phase extraction cartridges (Thermo Fisher Scientific, HyperSep Silica, silica bed 500 mg). Fractions were collected and lyophilized.

### Controlled Acid Hydrolysis of Laminaran

LOs were produced from laminaran by partial acid hydrolysis. In this study, response surface methodology (RSM) was used to optimize and investigate the influence of independent variables on the preparation of LOs from laminaran. Experiments were established based on a Box–Behnken Design (BBD) with three factors and three levels. The BBD design consisted of 17 experimental points, 5 replicates at the center of the design were used to allow for estimation of a pure error sum of squares. The partial acid hydrolysis of laminaran conditions include hydrolysis time (*X*_1_, 45–80 min), temperature for hydrolysis (*X*_2_, 60–80°C), and TFA concentration (*X*_3_, 0.75–1.25 mol/L). After partial acid hydrolysis, the reactant was immediately cooled in an ice bath to room temperature. The yield of LOs (include DP 2–5, mg/mL) was determined using high performance liquid chromatography and fitted to a quadratic polynomial model as follows formula:


$$\text{Y}=\beta_{0}+\sum \beta_{i}\,X_{i}+\sum \beta_{i\,i}\,X_{i}^{2}+\sum \beta_{i\,j}\,X_{i}\,X_{j}$$

where, Y is the predicted response (LOs yield mg/mL) associated with each factor level combination, *β_0_*, *β_*i*_*, *β_*ii*_*, and *β_*ij*_* are the regression coefficients for intercept, linear, quadratic, and interaction terms, respectively; *X*_*i*_ and *X_*j*_* are the independent variables.

The statistical analyses of data were carried out using BBD to establish the mathematical model, interpret the interaction and estimate the response of the independent variables. Experimental data were analyzed to fit a second-order polynomial model. The models were predicted through statistical analysis and regression analysis (ANOVA) using Design Expert V8. This software was also used to obtain the coefficients of the quadratic polynomial model. The quality of the fitted model was expressed by the coefficient of determination *R*^2^, and its statistical significance was checked with an *F*-test.

### High Performance Liquid Chromatography Analysis of Laminaran Oligosaccharides

High performance liquid chromatography (HPLC) analysis was carried out in an Alltech 1200 HPLC system (Alltech, United States) equipped with a LT-II evaporative light scattering detector (ELSD, Shimadzu Corporation, Japan). The analysis of sample was evaluated on a NH_2_ column (250 × 4.6 mm i.d., 5 μm). The column temperature was maintained at 35°C The eluent solutions were water (A) and acetonitrile (B) with a gradient solution of 75% B at 0–5 min, 75–55% B at 5–20 min, 55–40% B at 20–35 min, and 40% B at 35–40 min. The flow rate was 0.5 mL/min and the injection volume was 20 μL. ELSD conditions were as follows: temperature of drift tube was 50°C, the value of gain was 4, and the flow rate of nitrogen gas was 2 mL/min.

### Fast Protein Liquid Chromatography Purification of Laminaran Oligosaccharides

LOs were separated and purified by FPLC using a modified method as described previously (15). FPLC analysis was carried on an Akta FPLC system (AKTA Pure, GE Healthcare, Uppsala, Sweden) coupled with a refractive index detector (Agilent Technologies, Palo Alto, CA, United States). The LOs (0.5 g) were dissolved in distilled water (1 mL), and filtered using a 0.45 μm membrane before injected into the GE Healthcare column XK 16/100 packed with Sephadex LH-20. The LOs with different fractions were eluted with water at a flow rate of 0.5 mL/min. The collection volume was 3 mL per tube. The liquid corresponded to a single peak was collected for further analysis. The rest were concentrated and further purified using Bio-Gel P-2 extra fine.

### Electron Spray Ionization-Mass Spectrometry

The molecular masses of purified oligosaccharide with different degree of polymerization were measured using a Thermo TSQ Endura liquid chromatograph–mass spectrometer system (LC–MS system, Thermo Fisher Scientific, United States). Ionization was carried out using electrospray ionization (ESI) with a positive ion mode. The ionization mode was performed using the following conditions: temperature 300°C, source voltage 3.5 kV, drying gas N_2_ of 0.7 L/min, nebulizer pressure 25 psi, isolation width 4, fragment amplification 1.5, and masses scanned from 50 to 2,000 *m/z*. Before ESI-MS analysis, samples were prepared as previously described method (18). In brief, freeze-dried samples (10 mg) were submersed in 1 mL of acetonitrile/water solution (5%, *v/v*), and filtered through a 0.45 μm membrane.

### Nuclear Magnetic Resonance Analyses

Each purified oligosaccharide sample (50 mg) with different degree of polymerization was loaded into nuclear magnetic tube and dissolved in D_2_O at room temperature. The ^1^H spectrum and ^13^C nuclear magnetic resonance (NMR) spectrum were recorded on a Bruker AVANCE 500 spectrometer (Bruker BioSpin Corporation, Billerica, MA, United States).

### Antioxidant Activities of Laminaran Oligosaccharides

The DPPH radical scavenging activity of LOs was investigated by a previous method with slight modification (19). In brief, 100 μL different concentrations (0.1–2.0 mg/mL) of LOs were added to 100 μL of 50 mmol/L DPPH in ethanol. Then the mixture was shaken and incubated in dark place for 30 min, and the value of absorbance was measured at 517 nm. Ascorbic acid was used as a positive control. The ability of DPPH scavenging was calculated using the following equation:


DPPHscavengingactivity(%)=[1-(A-iA)j/A]0×100%

Where A_0_ was the absorbance of blank (ethanol instead of sample), A_*i*_ was the absorbance of sample (sample with DPPH-methanol solution), A_*j*_ was the absorbance of control (sample without DPPH-methanol solution).

The potential of LOs in scavenging ABTS radicals was evaluated as described earlier with some adjustments (20). Briefly, ABTS radical solution was prepared as follows: 7 mmol/L of ABTS solution (5 mL) was incubated with 2.45 mmol/L of potassium persulfate (88 μL) at room temperature for 16 h. Next, 2 mL ABTS radical solution were added into LOs samples (1 mL) with various concentrations (0.0625, 0.125, 0.25, 0.5, 1, and 2 mg/mL), respectively. After rection at room temperature (in dark place) for 10 min, the value of absorbance was measured at 734 nm. Ascorbic acid was used as the positive control. The ABTS radical scavenging activity (%) was calculated as DPPH radical scavenging activity equation above.

### Prebiotic Effect of Laminaran Oligosaccharides

The prebiotic effect of the LOs on *Bifidobacterium adolescentis* and *Lactobacillus plantarum* were carried in batch cultures according to previous study with minor modification (21). Tubes containing the basal MRS medium were supplemented with LOs, inoculated with *B. adolescentis* and *L. plantarum*, and incubated at 37°C for 48 h under anaerobic conditions. For comparative purposes, additional experiments were performed with media containing fructo-oligosaccharide (FOS) as positive control. The growth of each strain was monitored by measuring the optical density (OD) of the cultures at 600 nm. The OD_600_ values (expressed as mean ± *SD*, *n* = 3) were measured using a spectrophotometer.

## Results and Discussion

### Optimization of Hydrolysis Conditions

**FIGURE 1:**
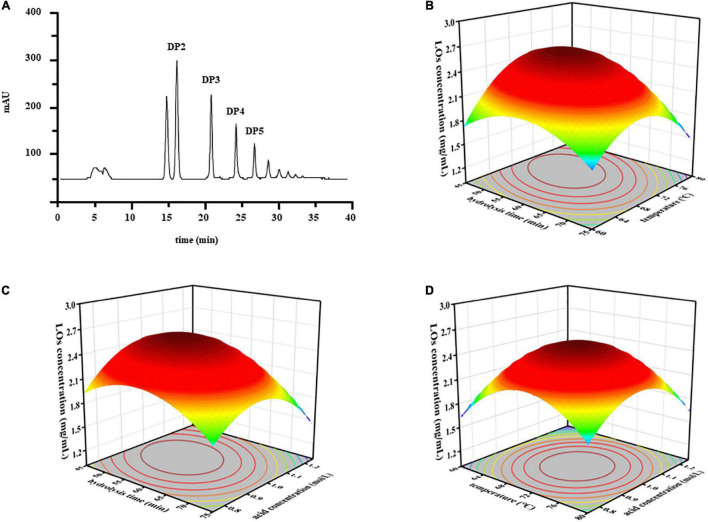
**(A)** The chromatograms of LOs separated by HPLC-ELSD. The response surface plots of the interaction effects of independent variables on the LOs production. **(B)** Hydrolysis time and temperature; **(C)** hydrolysis time and acid concentration; **(D)** temperature and acid concentration.

Response surface methodology (RSM) is an effective collection of statistical analysis methods that can be used to optimize different environmental processes and evaluate the effects of multiple variables, which helps reduce the process parameters with a minimum number of experiments. The yield of LOs was determined by HPLC-ELSD (Figure 1A). ANOVA was used to examine the significant variables and fitness of the regression model. The BBD results are listed in Table 1. Based on these results, the model *P*-value (possibility) was < 0.0001, indicating that the model was significant for responses in partial hydrolysis yield. The model *F*-value of 127.53 implies that there is only a 0.01% chance of occurrence owing to noise. The *P*-value of the lack-of-fit and *F*-value were 0.059 and 0.9787, respectively, which demonstrated that the lack-of-fit was insignificantly relative to the pure error and confirmed the validity of the model. Furthermore, the regression model exhibits a high degree of correlation between the experimental and predicted values. The coefficients of determination *R*^2^ and adjusted *R*^2^ were 0.9939 and 0.9861, respectively, whereas a relatively low coefficient of variation (2.60%) indicated better precision and reliability of the experiments.

**TABLE 1:** The box Behnken design with three independent variables, include hydrolysis time, temperature, and acid concentration for the LOs production.

Hydrolysis time (min)	Temperature (°C)	Acid concentration (mol/L)	LOs concentration (mg/mL)
45	60	1.00	1.72
75	60	1.00	1.65
45	80	1.00	2.20
75	80	1.00	1.43
45	70	0.75	1.91
75	70	0.75	1.75
45	70	1.25	2.05
75	70	1.25	1.43
60	60	0.75	1.61
60	80	0.75	1.76
60	60	1.25	1.56
60	80	1.25	1.62
60	70	1.00	2.55
60	70	1.00	2.62
60	70	1.00	2.57
60	70	1.00	2.56
60	70	1.00	2.44

The quadratic equation model estimated by observed data was as follows: LOs yield (mg/mL) = 2.55 − 0.20 *X*_1_ + 0.059 *X*_2_ − 0.046 *X*_3_ − 0.18 *X_1_X_2_* − 0.12 *X_1_X_3_* − 0.022 *X_2_X_3_* − 0.33 *X*_1_^2^ − 0.47 *X*_2_^2^ − 0.44 *X*_3_^2^, where *X*_1_, *X*_2_, and *X*_3_ denote hydrolysis time, temperature, and acid concentration, respectively. To determine the optimum levels of each variable for maximum yield of LOs, RSM plots were constructed using two independent variables. The relationships between LOs production and the three different variables (hydrolysis time *X*_1_, temperature *X*_2_, and acid concentration *X*_3_) using RSM are shown in Figures 1B–D. Based on the results, the predicted optimum yield of LOs (2.59 mg/mL) was computed as hydrolysis time of 54.84 min, temperature of 71.26 °C, and acid concentration of 1.00 mol/L. Considering the error from the actual operation, the experiment was conducted under these modified optimal conditions: hydrolysis time of 55 min, temperature of 71°C, and acid concentration of 1.00 mol/L, and LOs were obtained in a yield of 2.59 mg/mL. The controlled acid hydrolysis of laminaran seems to be the best choice for LOs production on a large scale because of its simplicity, affordability, and reproducibility.

### Selection of Size-Exclusion Chromatography Resins for Purification

FPLC is a form of preparative liquid chromatography that is often used to purify mixtures of proteins. It has advantages of high resolution, high automation (including auto sampler and peak collection), gradient program control, and availability of stationary phases in most common chromatography modes. Although the method was originally developed for purification of proteins, it has also been widely applied in separation and purification of other kinds of samples, such as RNA, oligonucleotides, polysaccharides, oligosaccharides. An appropriate size-exclusion chromatography (SEC) resin is the key parameter for obtaining high-purity oligosaccharides with varying DPs. Generally, Sephadex G-10, Sephadex LH-20, and Bio-Gel P-2 are commercially available and are used for the separation of carbohydrates with molecular weights in the range of 150–2,000 Da. Sephadex G-10 and LH-20 are beaded gel filtration media prepared by cross-linking dextran, which has been reported to yield excellent separation results for hyaluronan oligosaccharides (22) and arabinoxylan oligosaccharides (23), respectively. Kumar et al. separated fructo-oligosaccharides from onions using Bio-Gel P-2 (24). Therefore, the performance of these three different SEC resins (Sephadex G-10, Sephadex LH-20, and Bio-Gel P-2) for the purification of LOs from crude oligosaccharides was investigated and compared, and Bio-Gel P-2 was screened as a favorable resin. Enrichment of purified DP 2–5 from crude LOs exhibited excellent purification performance using Bio-Gel P-2.

### Identification of Laminaran Oligosaccharides

**FIGURE 2:**
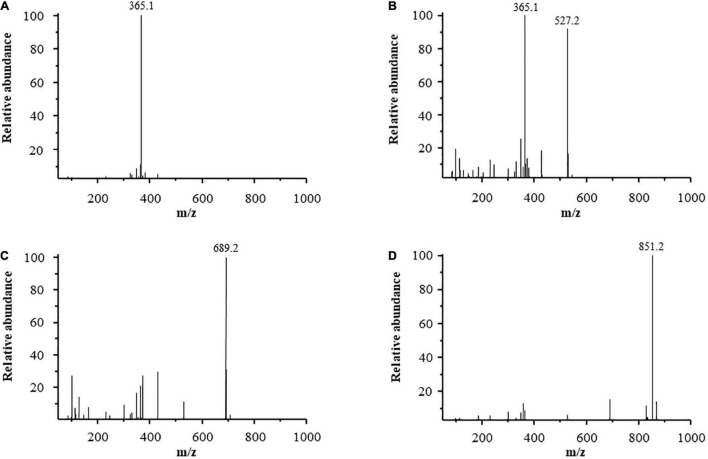
ESI-MS of purified LOS, **(A)** laminaribiose, **(B)** laminaritriose, **(C)** laminaritetrose, **(D)** laminaripentaose.

Mass spectrometry has been proven to be a sensitive, fast, and effective technique for analyzing the structure of oligosaccharides. This technique can provide detailed oligosaccharide structure information, such as molecular weight, monosaccharide composition, linkages, fragment patterns, and location of chemical modifications of the oligosaccharides (25, 26). Purified LOs were subjected to ESI-MS analysis. Figure 2 shows the ESI-MS profiles of the purified LOs. In the spectrum, peaks were observed that showed the [M + Na]^+^ ions at *m/z* 365.1 (Figure 2A), *m/z* 527.1 (Figure 2B), *m/z* 689.2 (Figure 2C), and *m/z* 851.2 (Figure 2D), corresponding to DP 2, DP 3, DP 4, and DP 5 lamirabiose-derived oligosaccharides, respectively. A similar mass spectra result was reported by Yang et al., with the hydrolysis products of laminaritriose plus Na at m/z 527 and laminaritetraose plus Na at m/z 689 (27).

Structural information at the molecular level is directly related to the chemical structural properties and biological interaction modes. The structural aspects of the purified LOs were assessed using ^1^H NMR and ^13^C NMR spectroscopy. NMR is one of the most effective non-destructive methods. This method is commonly used for the structural analysis of oligosaccharides. NMR data provide information about the oligosaccharide structure, including the monosaccharide composition, presence of α-type or β-type carbohydrates, linkage features, and sequences of the monosaccharide units (28).

**FIGURE 3:**
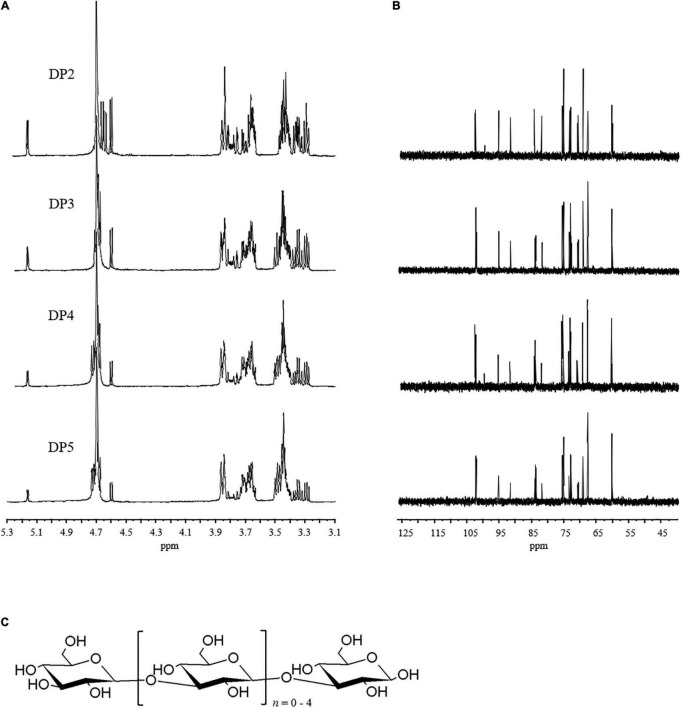
**(A)** ^1^H nuclear magnetic resonance (NMR) and **(B)** ^13^C NMR of the purified LOs. **(C)** Proposed structure of LOs.

The NMR spectra of the purified LOs are shown in Figure 3 and the chemical shifts of ^1^H and ^13^C signals are summarized in Table 2. The anomeric proton of non-reducing D-glucopyranosyl chemical shift at 4.60 ppm and anomeric carbon chemical shift is at 102.8 ppm, which denoted its β-anomeric form. The chemical shifts at 4.69 ppm were assigned to the H-1 and C-1 protons and signals of 1^→^3-linked-β-D-Glc*p*.

**TABLE 2:** ^1^H nuclear magnetic resonance (NMR) and ^13^C NMR chemical shifts of laminaran oligosaccharides DP2-DP5 (D_2_O, δ in ppm).

Residue	1	2	3	4	5	6
A	95.66–95.68/ 4.69–4.73	73.77–73.83/ 3.41–3.49	84.48–84.66/ 3.67–3.77	68.07–68.11/ 3.49–3.55	75.55–75.62/ 3.47–3.59	60.66–60.69/ 3.86–3.95; 3.75–3.84
B	102.48–102.51/ 4.68–4.78	73.36–73.45/ 3.38–3.46	84.23–84.27/ 3.43–3.46	68.08–68.11/ 3.43–3.4 6	75.57–75.63/ 3.49–3.59	60.66–60.69/ 3.84; 3.73–3.82

*A, →3)β-D-Glc; B, →3)-β-D-Glc-(1→3).*

The ^13^C NMR signals in the region in the range of 68.1–84.7 ppm were attributed to C-2^→^C-5 carbons of the pyranoid rings of 1^→^3-linked-β-D-Glc and β-D-Glc. In the spectrum of ^1^H NMR, a significant chemical shift in the range of 3.43–3.46 ppm was observed and assigned to H-3 of 1^→^3-linked-β-D-Glc*p*. It also included resonances characteristic of 1^→^3-linked-β-D-Glc and β-D-Glc such as signals from ring protons (H-2^→^H-5) in the range of 3.38–3.59 ppm. These data were in accordance with those of previous studies (29, 30), which indicated that laminaran is a non-reducing glucosyl end linked to various numbers of 1^→^3-linked-β-D-Glc*p*. The structure of laminaran derived from *Sargassum duplicatum* reported by Usoltseva et al. consists primarily of a linear sugar chain of 1^→^3-linked-β-D-Glc*p* residues with a terminal β-D-Glc*p* residue (31).

MS was used to determine the molecular weight of LOs while ^1^H and ^13^C NMR were employed to determine the sugar residue sequences. From the results of structural analyses, it can be concluded that the repeating units of LOs are 1^→^3-linked-β-D-Glc*p* with non-reducing terminal β-D-Glc*p* residues at C-3. The probable structures of the LOs are shown in Figure 3C.

### Antioxidant Effects of Different Purified Laminaran Oligosaccharides

Reactive oxygen species, including superoxide anions, hydrogen peroxide, and hydroxyl radicals are produced in cells through oxygen metabolism and function. In contrast, several reactive oxygen species can contribute to various oxidative stress-induced diseases such as diabetes, cancer, cardiovascular, and neurodegenerative diseases (32). Recently, oligosaccharides have been demonstrated to play an important role as free-radical scavengers and prevent oxidative damage in living organisms. Their ability strongly depends on chemical structures such as monosaccharide composition, type of glyosidic, and molecular weight (25). The *in vitro* experiments of antioxidant activities are generally determined through DPPH radical scavenging, lipid peroxide inhibition, ferric reducing antioxidant power, nitric oxide scavenging, and ABTS radical scavenging with the advantages of simplicity, cost effectiveness, and ease of interpretation. In this study, DPPH and ABTS radical scavenging assays were used to investigate the antioxidant activities of LOs.

**FIGURE 4:**
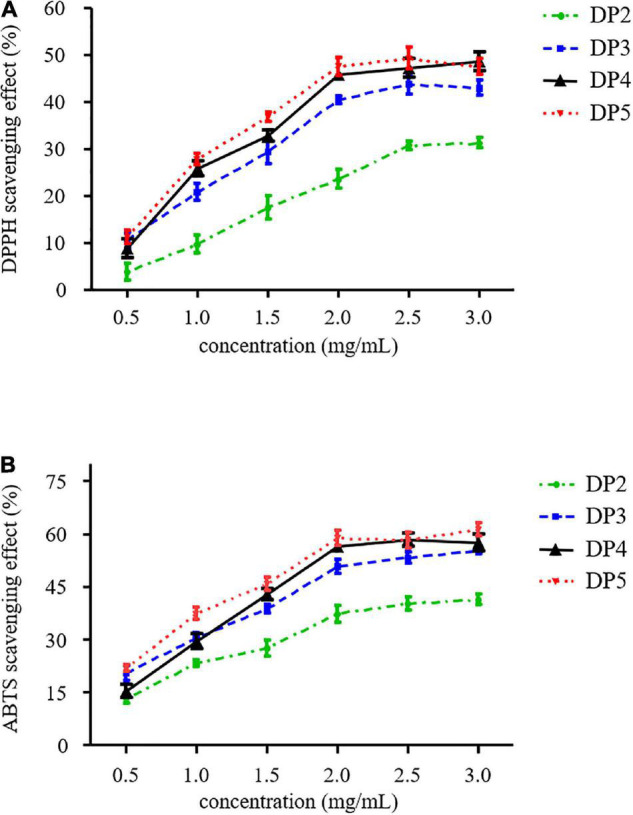
**(A)** DPPH scavenging effect and **(B)** ABTS scavenging effect of purified LOs.

DPPH is a stable radical that can be used to determine the antioxidant activity of LOs by measuring the decrease in absorbance at 517 nm. As shown in Figure 4A, the scavenging abilities of all the samples were concentration-dependent. The scavenging of the DPPH radical of oligosaccharides was due to their hydrogen-donating ability that led to the formation of a stable molecule (33). These results are similar to those of other neutral oligosaccharides produced from natural polysaccharides such as xylo-oligosaccharides and gluco-oligosaccharides. Kallel et al. reported that half-maximal inhibitory concentration (IC_50_) of xylo-oligosaccharides from garlic straw xylan was 0.45 mg/mL (33). In addition, IC_50_ of β-gluco-triose and β-gluco-tetraose hydrolysis from β-glucan was 1.8 mg/mL (34).

ABTS radical scavenging ability is another commonly used method in functional food research to measure antioxidant activity. Figure 4B illustrates that a correlation was established between LOs concentration and ABTS radical-scavenging ability. At low concentrations, this ability was enhanced rapidly with an increase in concentration; however, the rising trend slowed when the concentration of LOs was more than 2.0 mg/mL. The ABTS radical scavenging rates of DP2 and DP5 at a concentration of 2 mg/mL were 37.3 and 58.8%, respectively. DP3–5 exhibited higher ABTS radical scavenging activity, whereas DP2 exhibited relatively low ability. Therefore, the antioxidant activity of the LOs in this study may be attributed to the DP of the oligosaccharides.

The mechanism of the ABTS radical scavenging ability of oligosaccharides may be similar to that of the DPPH radical scavenging mechanism. In general, C-2 and C-6 hydroxyls of oligosaccharides are mainly involved in H-atom transfer reactions toward the ABTS radical (35). It is hypothesized that hydrogen abstraction occurs at C-2 or C-6 positions of LOs captured at these positions to yield a stable ABTS-oligosaccharide form. Furthermore, Kang et al. reported agaro-oligosaccharide production through enzymatic hydrolysis of agarose, where the scavenging activities of agaro-oligosaccharides against ABTS radicals ranged from 60.34 to 83.84% (35). Choi et al. reported that low-molecular-weight laminaran from laminaran degraded by gamma irradiation exhibited high antioxidant activities (7).

These aforementioned antioxidant activity results reveal that LOs are free radical inhibitors and primary antioxidants that scavenge free radicals. This indicates that LOs could potentially be exploited in the preparation of functional food products.

### Prebiotic Activities of Laminaran Oligosaccharides

**FIGURE 5:**
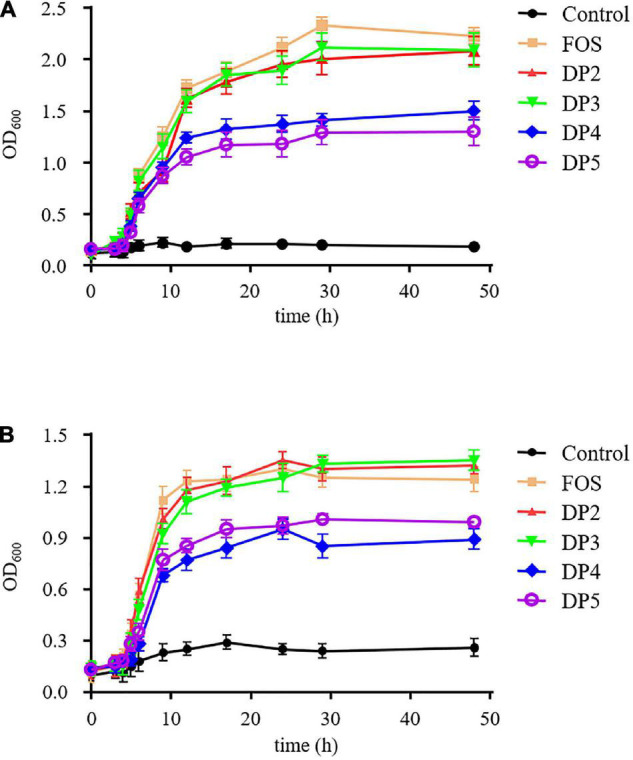
The growth curves for *B. adolescentis* **(A)** and *L. plantarum* **(B)** grown in basal medium supplemented with purified LOs. The growth curves for each strain with a range of test samples were determined by measuring the OD of the cultures at 600 nm intermittently over a 48 h period.

Prebiotics are mainly targeted to enhance the growth of beneficial bacteria, such as bifidobacteria and lactobacilli in the gut (12, 36). Therefore, we tested the potential prebiotic effects of LOs. The proliferation of probiotics in the media with different purified LOs as carbon sources is shown in Figure 5. *B. adolescentis* strains grown in FOS-, DP2, and DP3 supplemented media exhibited similar and rapid growth with high final OD 600 values; however, they grew significantly slower in DP4 and DP5 supplemented media. The results demonstrated that the varying DPs of LOs had different effects on the growth of *B. adolescentis* and *L. plantarum*. The growth of *L. plantarum* in DP4 and DP5 resulted in lower absorbance levels of 0.95 and 1.01, respectively. This lower level of growth is likely related to the DP of the LOs. A lower DP generally induced faster growth of the probiotics because the lower DP oligosaccharides were cleaved into monosaccharides as energy sources.

These findings are similar to those made by Zhang et al. who reported that neoagaro-oligosaccharides with DP3 exhibited the best growth stimulation for *Sterptococcus thermophilus*, and its OD_600_ reached 0.94 after 72 h of incubation. The order of prebiotic effects of neoagaro-oligosaccharides was DP3 > DP5 > DP8 (37). In this study, LOs exhibited beneficial effects on the growth of probiotic strains such as bifidobacteria and lactobacilli. Therefore, LOs could be regarded as prebiotics, which might have the same benefits as FOS.

## Conclusion

LOs were obtained using controlled acid hydrolysis of laminaran from *L. digitata*. BBD was applied to determine the optimum process parameters that afforded a high LOs yield. By analyzing the second-order polynomial model, a maximum LOs yield of 2.59 mg/mL was obtained under the following conditions: hydrolysis time of 55 min, temperature of 71°C, and acid concentration of 1.00 mol/L. Subsequently, large-scale pure LOs were automatically and efficiently separated and purified using the FPLC-RID technique, whereas the Bio-Gel P-2 size exclusion resin was suitable for LOs separation. These are useful for pharmaceutical application and for the qualitative and quantitative analyses of LOs as additives in health foods.

The obtained results clearly demonstrate that each LOs fraction exhibits antioxidant activity, which follows a concentration-dependent response for DPPH and ABTS radicals scavenging activities. In particular, DP5 exhibited excellent antioxidant capacity. Prebiotic experiments demonstrated the growth of *B. adolescentis* and *L. plantarum* in LOs. LOs with DP3 exhibited remarkable prebiotic activity. Although *in vitro* assessment of the functionality of oligosaccharides has some limitations, the results provide a direction for future research and application and improve our understanding of the potential applications of LOs. These results suggest that LOs exhibit antioxidant and prebiotic activities, indicating their potential application in functional foods aimed at improving human gastrointestinal health and preventing gut diseases.

## Data Availability Statement

The original contributions presented in this study are included in the article/supplementary material, further inquiries can be directed to the corresponding author.

## Ethics Statement

The protocol for these experiments was approved by the Ethics Committee of Guangdong Ocean University.

## Author Contributions

K-LC and SZ designed the research. K-LC conducted the research. K-LC, J-KL, and SZ analyzed the data. K-LC and J-KL wrote the manuscript. SZ edited the manuscript. All authors read and approved the final manuscript.

## Conflict of Interest

The authors declare that the research was conducted in the absence of any commercial or financial relationships that could be construed as a potential conflict of interest.

## Publisher’s Note

All claims expressed in this article are solely those of the authors and do not necessarily represent those of their affiliated organizations, or those of the publisher, the editors and the reviewers. Any product that may be evaluated in this article, or claim that may be made by its manufacturer, is not guaranteed or endorsed by the publisher.
